# Quality Preservation of Ready-to-Eat Prickly Pears by Peels Recycling

**DOI:** 10.3390/foods11142016

**Published:** 2022-07-07

**Authors:** Olimpia Panza, Valentina Lacivita, Amalia Conte, Matteo Alessandro Del Nobile

**Affiliations:** Department of Agricultural Science, Food and Environment, University of Foggia, 71121 Foggia, Italy; olimpia.panza@unifg.it (O.P.); valentina.lacivita@unifg.it (V.L.); matteo.delnobile@unifg.it (M.A.D.N.)

**Keywords:** prickly pears, fresh-cut fruit, fruit by-products, peel-enriched coating

## Abstract

In the current study, prickly pear peel was advantageously recycled to preserve fruit quality. Specifically, the investigated by-products were transformed into powder and then loaded into an alginate-based solution to be applied as coating to peeled prickly pears, to give an example of sustainable minimally processed fruit. For comparison, uncoated fruit, and coated prickly pears without any powder were also prepared. During storage at refrigerated temperature, coated and uncoated samples were tested for weight loss, microbial and fungal proliferation, as well as for sensory quality acceptance. The results were interesting because great differences were found between coated and uncoated fruit, in that coating the fruit delayed weight loss and spoilage, compared to uncoated fruit. Between the simple coating and the coating with peel powder, slight differences were recorded in favor of the peel-enriched coating. In fact, it allowed the promotion of better fruit preservation, and sensory quality. Therefore, prickly pear peels, that represent abundant by-products during prickly pear processing, could be advantageously recycled to preserve fruit quality.

## 1. Introduction

The growing demand for minimally processed fruit and vegetables is related to safety, nutritional quality and sensory properties of fresh products. In addition to health benefits, rapidly changing lifestyles, offering significantly less time for planning and preparing of meals, have promoted great expansion of demand for ready-to-eat fruit and vegetables [1,2]. However, washing, peeling and cutting promote faster physiological deterioration of minimally processed produce. Important biochemical changes and microbial proliferation can provoke significant shelf life reduction of fresh-cut products, compared to whole fruits [3].

The prickly pear cactus (*Opuntia ficus-indica*) is cultivated in several countries, including Mediterranean regions. In the literature, many studies show that these fruits can be considered a source of nutritional compounds [4]. In particular, several authors have reported that prickly pears contain appreciable quantities of soluble fibers and antioxidant compounds, such as ascorbic acid, phenols, flavonoids [5]. Prickly pears are rich in pigments of betalain derivatives, such as betacyanin, responsible for the fruit’s purple red color, or betaxanthin, responsible for its yellow-orange color [6]. Both these compounds exert beneficial effects on cell growth and inflammation [7]. Prickly pears are also a good source of minerals, particularly calcium, magnesium, potassium and phosphorus [8,9]. Prickly pears, highly appreciated for their flavor, sweetness and juiciness, are commonly consumed fresh because they are perishable fruit [10]. In fact, these fruits are characterized by a high quantity of water, low acidity and high sugar content [11], thus becoming ideal substrates for microbial proliferation.

The scientific literature reports some information dealing with the techniques to store minimally processed prickly pears. Specifically, the main strategies are based on modified atmosphere packaging [12], proper storage temperature [11] or specific packaging film [13]. The edible coatings are also valid tools to control peeled fruit quality [14,15]. Coatings gained considerable attention for extending the shelf life of fresh-cut fruits [16], as they are able to act as a barrier to water vapor and gas, to reduce the rate of moisture loss, and the fruit’s oxidative reactions [17,18,19,20,21]. Liguori et al. [22] treated minimally processed cactus pear fruits with a mucilage-based coating extracted from *Opuntia ficus-indica* cladodes. Results showed that the mucilage-based coating improved the quality and preserved the nutraceutical value of peeled fruits.

From a more sustainable perspective, the recycling of prickly pear peels as a source of natural active ingredients is proposed to prolong the shelf life of peeled fruit. The peels of prickly pears (almost half of the fruit) represent an environmental problem. It is imperative to develop effective strategies to valorize these by-products that are generally discarded. One of the most interesting research approaches is the production of high added value products containing by-products. In fact, due to the high content of bioactive compounds, dietary fiber, fatty acids, lipid classes, sterols, fat-soluble vitamins and b-carotene, abundantly reviewed in the literature [23,24,25,26,27], prickly pear peels could be conveniently used as functional ingredients in some food preparations, such as bakery products [28,29]. The beneficial health effects of consuming healthy dietary patterns rich in dietary fiber from whole plant foods include improving gut health, lowering elevated LDL-cholesterol, reducing the risk of excessive weight gain and obesity, decreasing cardiovascular disease, coronary heart disease and mortality risks, reducing risks of several cancers, stroke and type 2 diabetes and improving the odds for successful aging [30,31]. Due to the high content of bioactive compounds with antimicrobial and/or antioxidant properties, extracts from peels of prickly pears were also adopted for food preservation. In this context, margarine [32] and sliced beef [33] can be cited as successful food product case-studies. The research of Dilucia et al. [34] is a rare example of peels completely recycled to prolong the shelf life of fresh fish burgers, according to the zero-waste concept.

Considering the potential of by-products in the food sector and the great advantage of their complete recovery as new ingredients, the current study proposed recycling prickly pear peels to control the detrimental phenomena that occur during the storage of the fresh-cut fruit. Therefore, the effects of an eco-friendly coating, made up of alginate and peel powder, were assessed on both microbiological stability and sensory quality of the peeled fruit. For comparison, peeled prickly pears without any coating and fruit stored with a simple alginate coating were also considered.

## 2. Materials and Methods

### 2.1. Fruit Peeling and Coating

Red prickly pears (*Opuntia ficus-indica* (L.) Mill.), cultivar Sanguigna, were kindly provided by a local dealer (Manfredonia, Puglia, Italy), transported to the laboratory and stored at 4 °C prior to processing. The fruits were carefully washed in water, then dipped into chlorinated water (20 mL/L) for 5 min, rinsed again and, finally, air dried. After cleaning, the fruits were manually peeled. The peels were dried at 37 °C in a vacuum dehydrator (Melchioni-Babele, Milan, Italy) and then milled to obtain a fine powder (<500 µm) that was completely utilized, without leaving any further waste.

For the coating solutions food-grade sodium alginate (Farmalabor, Canosa, Italy 5% *w*/*v*) was dissolved in sterile water. By heating on a stirring hot plate for 15 min at 70 °C, the alginate solution became clear. Two different coating solutions were prepared: a solution containing prickly pear peel powder (7.5 g/400 mL of solution) and a control one without any powder. For preparing coated fruit, each peeled prickly pear was dipped in the peel-enriched, or in the control, solution for 1 min. Then, to promote the alginate gel forming, each coated fruit was submerged in a calcium chloride solution (5% *w*/*w*) for 2 min. Two coated fruits were placed in a food tray with a pad and packaged in air using a commercial food bag made up of polyethylene (thickness = 25 µm). For comparison, uncoated peeled fruits were also prepared and packaged (Ctrl). All the samples were kept under refrigeration (4 ± 1 °C) for 10 days. Samples with control coating were named Coat C and fruits with the peel-enriched coating were named Coat A.

### 2.2. Microbiological Analyses and pH Determination of Prickly Pears

During the entire storage period, uncoated and coated samples were tested for microbial contamination. To this aim, 10 g of each sample were aseptically weighed in a sterile stomacher bag, diluted with physiological water (dilution 1:10) and homogenized for 120 s. Subsequently, decimal dilutions were made, then plated on specific media in Petri dishes to enumerate different microbial groups. Plate Count Agar (PCA, Oxoid, Milan, Italy) incubated at 30 °C for 48 h and 5 °C for 10 days for mesophilic and psychrotrophic bacteria, respectively; de Man Rogosa Sharpe Agar (MRS, Oxoid, Milan, Italy), supplemented with cycloheximide (0.1 g/L Sigma, Milan, Italy) incubated at 37 °C for 48 h for lactic acid bacteria; Sabouraud Dextrose Agar, added with 0.1 g/L of chloramphenicol (C. Erba, Milan, Italy), incubated at 25 °C for 48 h for yeasts and at 25 °C for 5 days for molds. The microbiological analyses were conducted twice on two different samples. The results are expressed as log cfu/g. The microbial thresholds were set at 5 × 10^7^ cfu/g for total viable mesophilic and psychrotrophic bacteria [3] and 10^6^ cfu/g for yeasts [20]. By fitting the experimental data, the microbiological acceptability limit (MAL), was calculated. This parameter has to be intended as the storage period (days) to reach the microbiological threshold. It was calculated according to the same mathematical approach (re-parameterized version of the Gompertz equation) already reported in the study of Costa et al. [3], also dealing with the shelf life of fresh-cut produce.

The pH measurement was performed in duplicate, on two different samples, on the first homogeneous dilution of prickly pears, by a pH meter (Crison, Barcelona, Spain).

### 2.3. Weight Loss and Moisture Content of Prickly Pears

Prickly pear fruits were weighted during the storage time to calculate the percentage weight loss:

WL (%) = [(Wi − Wf)/Wi] × 100(1)

where: WL (%) is the percentage weight loss at time t, Wi is the initial sample weight and Wf is the sample weight at time t. Coated cactus pears were weighted with their coating. A digital technical balance (± 0.1 g) (Gibertini Europe, Milan, Italy) was used. Two repetitions were performed for each measurement, using two different samples.

The moisture content of prickly pear samples was determined on 5 g according to the AOAC methods [35]. Moisture (percentage) was expressed as g water/100 g of dry matter.

### 2.4. Chemical Analyses of Prickly Pears

The following analytical grade reagents were used: Folin–Ciocalteu reagent, gallic acid monohydrate, methanol, hydrochloric acid, 2,2-azino-bis(3-ethylbenzothiazoline-6-sulfonic acid) diammonium salt (ABTS), potassium persulfate, Trolox (6-hydroxy-2,5,7,8-tetramethylchroman-2-carboxylic acid), aluminium chloride, sodium nitrite, sodium hydroxide solution, and quercetin, supplied from Sigma-Aldrich (Milan, Italy); anhydrous sodium carbonate from Carlo Erba (Milan, Italy). To determine total phenol content (TPC), total flavonoids (TFC) and antioxidant activity (ABTS) of peels and fresh-cut fruit products, the method of Mahloko et al. [36], with slight modifications, was carried out for sample extraction. In particular, the extracts were prepared mixing 2 g of each sample, previously dried at 35 °C for 24 h, with 20 mL of acidified methanol (80% MeOH in H_2_O acidified with 1% HCl). The mixtures were included in 50 mL centrifuge tubes for 2 h at 60 °C (agitation at 83 rpm). Subsequently, the mixtures were centrifuged for 20 min at 5000 rpm at 25 °C and the supernatants were separated and used for the analyses. The chemical analyses were performed at the beginning and at the end of the observation period. For the TPC, the Folin-Ciocalteu procedure was used [37]. Specifically, 0.5 mL of extract was mixed with 2.5 mL of Folin-Ciocalteu reagent and after 5 min 2 mL of Na_2_CO_3_ (4%) were added. The sample was kept in the dark at room temperature for 2 h. The absorbance was measured at 740 nm by a UV-vis spectrophotometer (UV1800, Shimadzu Italia s.r.l., Milan, Italy). TPC was quantified from a calibration curve (3.12–100 mg/L; R^2^ = 0.999) using a standard solution of gallic acid. TPC was expressed as mg of gallic acid/g of dry weight. TFC in the prickly pear extracts was determined with the colorimetric method of aluminum chloride, according to the procedure of Huang and Ho [38], with some modifications. Specifically, the extracts (0.5 mL) were mixed with 2 mL of distilled water and 150 μL of a 5% sodium nitrite solution. After 6 min, 150 μL of a 10% aluminum chloride solution was added and the mixture was allowed to stand for other 6 min. Then, 1 mL of 1 M sodium hydroxide and 1.2 mL of distilled water were added to reach the total volume of 5 mL. Then, the solutions were mixed and, for each sample, the absorbance was read in triplicate at 415 nm. The standard curve was prepared using quercetin as the standard in a proper range (6.25–400 mg/L; R^2^ = 0.995) and the total amount of flavonoids was expressed in mg of quercetin/g of dry weight. The antioxidant activity was assessed using the ABTS test, according to the method by Re et al. [39]. After obtaining the diluted solution of ABTS^+^ from the stock solution with the addition of potassium persulfate, 200 μL of sample extract was added to 2 mL of diluted ABTS solution and, after 6 min at 30 °C, the mixture was measured at 734 nm. A calibration curve was built using Trolox as the standard, at concentrations between 12.5 mg/L and 500 mg/L (R^2^ = 0.990). The antioxidant activity was expressed as mg Trolox equivalents/g of dry weight. All chemical analyses were made in triplicate.

### 2.5. Sensory Analyses of Prickly Pears

During storage, a panel of 7 trained members assessed the fruit sensory quality. Specific sensory parameters were considered as odor, color and texture, as well as fruit overall quality [35]. The judges were members of the laboratory, with various years of experience in sensory evaluation of fresh-cut fruit before the current study. Anyhow, prior to this evaluation, two sections (1 h/section) were carried out to adjust the members and define the sensory evaluation form. For the analysis, a 9-point scale was used where 9 corresponded to “like very much” and 1 to “dislike very much”, according to a similar procedure previously reported in the literature for fresh-cut melon [40]. A score equal to 5 was intended as the threshold for fruit acceptability. Considering that overall quality values can be intended as an average among the different tested attributes, the fitting of these experimental data allowed quantifying the sensory acceptability limit (SAL), which represents the number of days necessary to reach the threshold score (5). SAL values were calculated according to the fitting procedure (re-parameterized version of the Gompertz equation) also reported by Costa et al. [3].

### 2.6. Statistical Analyses

In order to statistically compare the fitting parameters, (MAL and SAL), the one-way ANOVA analysis was used. To determine significant differences among samples the Duncan’s multiple range test, with the option of homogeneous groups (*p* < 0.05), was adopted. Statistica 7.1 for Windows (StatSoft Inc., Tulsa, OK, USA) was used for data elaboration.

## 3. Results and Discussion

**Figure 1:**
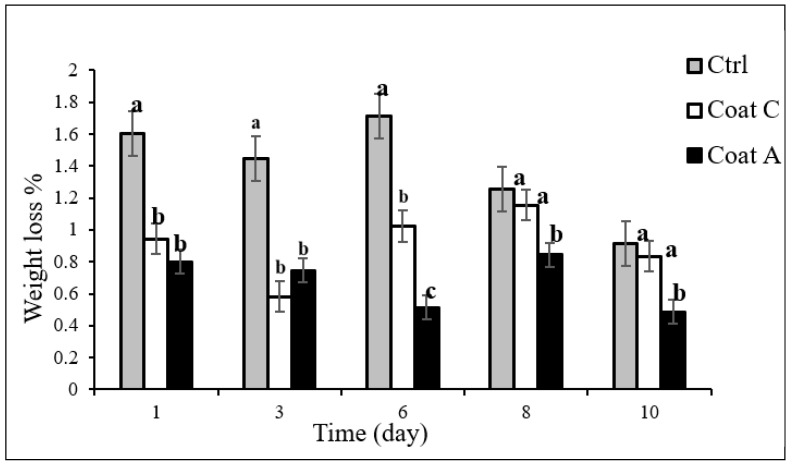
Weight loss (%) of prickly pears during storage at 4 °C. ^a,b,c^ Different superscript letters indicate that means are significantly different (*p* < 0.05). Ctrl = sample without coating; Coat C = sample with alginate coating; Coat A = sample with alginate and peels.

Prickly pear samples were compared for weight loss during refrigerated storage. This is an important parameter that may affect the quality of minimally processed fresh commodities [41] (Figure 1). The reduction in weight was very low during the entire experiment, with a maximum of 1.7%. Data from literature recorded on prickly pears stored under similar storage regimes also confirmed this trend. Specifically, Piga et al. [11] verified that minimally processed fruit stored at 4 °C showed a weight loss threefold lower than fruit at 15 °C; the reduction in weight was very low, with a maximum of 0.66 g/100 g after 8 d at 15 °C. Similarly, in another paper also dealing with minimally processed cactus pear, Piga et al. [42] verified that the reduction in weight was slight during the entire experiment, with a maximum of 0.15 g/100 g after 9 days at 4 °C. Liguori et al. [22] verified that control samples of *Opuntia ficus-indica* samples showed a weight loss from 2 to 2.5 times higher than coated samples during cold storage, and differences between coated and uncoated fruit were significant starting from day 1 until the end of the cold storage period (9 days).

Some differences can be highlighted among samples. In particular, two trends were found, one for the first week and another one for the last days of observation. Specifically, looking at data until the 6th day, it’s possible to observe that the control fruit presented weight loss of about 1.5%, whereas, the two coated prickly pears presented weight loss in the range of 0.5–1%. For the last two sampling times, the control prickly pears experienced weight loss of around 1%, similar to that of the simply coated fruit (Coat C). The fruits with the peel-enriched coating (Coat A) experienced significantly lower (*p* < 0.05) weight loss than the Coat C prickly pears and the control prickly pears. Wan et al. [16], in a recent review, highlighted the potential of edible films and coatings applied to fresh-cut fruit, not only against microbial decay but also to prevent physiological changes and physical loss during storage. Wong et al. [19] also found that apple slices without coating showed the greatest amount of weight loss after 7 days of storage. Manzoor et al. [20] also demonstrated that a control sample of sliced kiwi showed the greatest percentage weight loss, compared to nano-emulsion coated fruit. Edible coating barrier properties to prevent weight loss are well known in the literature. Moreover, peels forming the coating further promote fruit preservation from weight loss.

**Figure 2:**
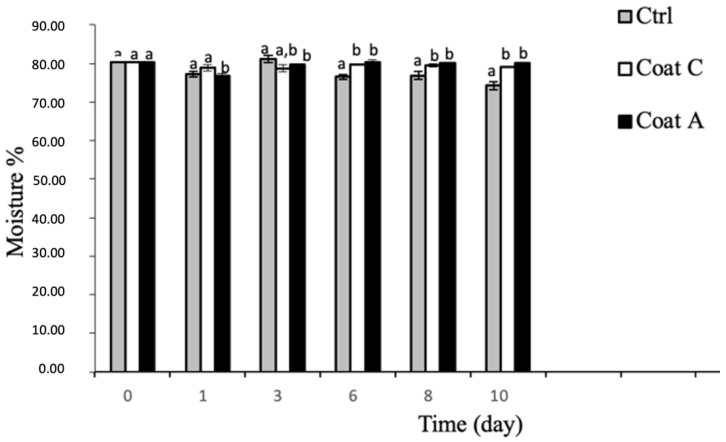
Moisture content (percentage) in the control and coated prickly pears stored at 4 °C. ^a,b^ Different superscript letters indicate that means are significantly different (*p* < 0.05). Ctrl = sample without coating; Coat C = sample with alginate coating; Coat A = sample with alginate and peels.

Data regarding moisture are in line with experimental findings recorded in terms of weight loss (Figure 2). In fact, for the first three monitoring times, control and coated fruit were very comparable, with moisture accounting for about 80%. After the first 6 days, the control and coated fruit differed significantly (*p* < 0.05). In particular, the moisture drop was more marked in the uncoated fruit, compared to coated samples. Between the coating with and without, peels slight differences could be observed, even though they were not statistically significant (*p* > 0.05). It was found that moisture content in samples with peel-enriched coating was slightly higher than the simply coated fruit, for almost the entire observation period.

**Figure 3:**
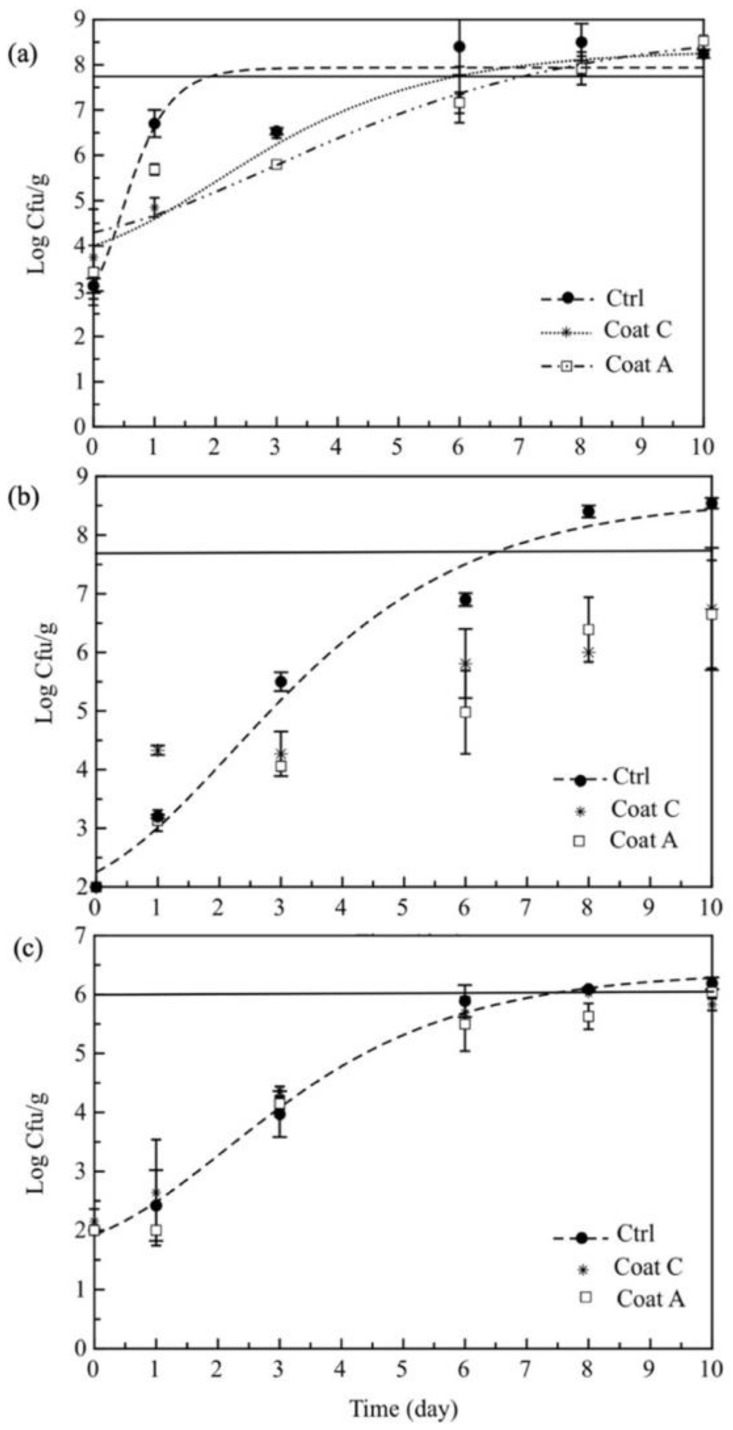
Mesophilic (**a**), psychrotrophic bacteria (**b**) and yeasts (**c**) in control and coated prickly pears stored at 4 °C. Data are presented as mean ± standard deviation. Curves are the fitting to the experimental data. The line indicates the threshold. Data not reaching the threshold were not fitted. Ctrl = sample without coating; Coat C = sample with alginate coating; Coat A = sample with alginate and peels.

Microbial proliferation in coated and uncoated samples was quite different (Figure 3). As regards to mesophilic bacteria, a rapid evolution of viable cell concentration could be observed in the uncoated fruit compared to coated samples, that were characterized by a slower kinetic, and a more marked, lag phase. Therefore, while the control fruit became unacceptable within a few days, crossing the threshold value (5 × 10^7^ cfu/g), both coated samples remained acceptable for more time. Comparing these last two samples, it was possible to see that fruit peel in the coating further promoted a delay in the growth kinetic of mesophilic bacteria.

Regarding psychrotrophic bacteria (Figure 3b), important differences were found among the samples. Specifically, while prickly pears without coating remained acceptable for less than a week, coated fruit never reached the set threshold (5 × 10^7^ cfu/g). Comparing the two coated fruit, it was interesting to observe that, for samples in contact with the coating containing the peels, a certain delay in the microbial proliferation could be underlined in the first week of storage.

Yeasts grew in all the samples with a very similar kinetic (Figure 3c); however, while the control fruit reached the threshold, both the coated prickly pears never went beyond the yeast limit (10^6^ cfu/g). As regards prickly pears with peels in the coating, as often as not, it was possible to see slightly lower yeast count than for the other tested fruit samples.

Microbial data recorded for all the spoilage groups were in line with other literature data. In fact, significant proliferations of bacterial and fungal counts were found in prickly pears stored at 4 °C under passive modified conditions, most probably due to the low acid content of this pulp [43]. Palma et al. [43] verified that fruit cultivars can be responsible for different microbial evolution during storage. As a fact, Díaz-Delgado et al. [13] very recently found low microbial and fungal proliferation in white and orange *Opuntia ficus-indica* fruits from the Canary Islands (Spain) peeled in different ways (by hand or with an electric peeler), packed in two micro-perforated films with different permeability and then stored at 7 °C. Del Nobile et al. [15], who also studied minimally processed prickly pears (*Opuntia ficus indica*, cv. ‘Gialla’), found a rapid microbial proliferation in fresh-cut fruit with and without coating, packaged in bio-based polymeric systems and stored at 4° C. The fungal and microbial proliferation recorded in the various packaged samples highlighted the positive effects of peels and confirmed the antimicrobial properties of these by-products, abundantly recognized in the literature [23,24,25,26,27].

In order to quantitatively compare our experimental data, a mathematical approach was used [3]. The model parameters (MAL) obtained from the fitting procedure are reported in Table 1. As can be seen in the first column, MAL for mesophilic bacteria of control fruit was about 4 days, whereas MAL for both coated samples were more than 6 days. In the second and third columns of the same table, results for psychrotrophic bacteria and yeasts are listed, respectively. In this case control prickly pears lasted more than 6 and 7 days, for psychrotrophic bacteria and yeasts, respectively, whereas both coated fruits remained acceptable for the entire storage period and, therefore, MAL higher than 10 days were indicated.

Comparing the results from the fitting procedure, it was possible to infer the microbiological shelf life of our samples, which is the time for products to remain acceptable with regards to microbial and fungal spoilage (Table 1). It is interesting to observe that mesophilic bacteria are the microbial population that greatly influenced product shelf life and, therefore, prickly pears remained acceptable for about 4 days when stored without any coating and for more than 6 days when a coting was applied. For the coating containing fruit peels a slight increase (*p* > 0.05) in the microbiological shelf life could be observed (6.79 vs. 6.41) if compared to the simply coated fruit. However, probably the by-product concentration was not so high as to promote very marked microbial control.

Lactic acid bacteria and molds were also monitored, but their concentrations remained in an acceptable range within the microbiological shelf life. Table 2 reports specific experimental data recorded during storage for each sample. As can be seen, the growth kinetics of lactic acid bacteria were similar among the three types of samples; all the prickly pears started from low viable cell concentrations (around 10^2^ cfu/g) and proliferated very slowly. As regards molds, very low counts (around 3 log cfu/g) were detected in both coated fruits during the entire storage period, whereas the values increased from 2 to 6.45 log cfu/g after 10 days of storage in the uncoated prickly pears. These data are in line with literature evidence; other authors also highlighted low contamination of fresh-cut prickly pears by lactic acid bacteria [15] and molds [11,43].

To sum up, findings recorded on all the fungal and microbial groups confirmed the ability of coating to control the spoiling of the fruit. Del Nobile et al. [15] also verified that an alginate-based coating, applied on half-peeled prickly pears, promoted microbiological shelf life prolongation from 9 to 13 days, whereas, agar or hydrogel were found not to be appropriate coating for the fruit as fresh-cut produce. Peels, in particular, represent valid ingredients for the microbiological stability of prickly pears, even though their inclusion in the polymeric network made up of alginate limited their effects. A direct application of peels to fruit would probably amplify their activity, as demonstrated in the case study of Dilucia et al. [33], where these by-products were added to fish burger formulation and were found to be very effective in controlling microbial proliferation.

Data on pH recorded at each sampling time are reported in Table 3. Looking at the data, similar trends could be observed in the three samples during the 10 days of storage. In particular, pH ranged between 5.7 and 4.8 in all the samples, with slight fluctuations that could be ascribed to the microbial proliferation occurring in the fruit. Del Nobile et al. [15] also found the pH of prickly pears changing from 5.5 to 4.75 during storage. Data suggested that there was no influence in terms of either the coating nor peel powder incorporated in the alginate polymeric system on evolution of the pH.

Peel before application and fruit samples during cold storage were analyzed for chemical quality. In particular, the content of polyphenols and flavonoids, as well as antioxidant properties, were assessed on the peel as it is, and on the peeled fruit at the beginning and at the end of the storage time. As regards peels, the following contents were recorded: a content of polyphenols equal to 12.20 ± 0.19 mg of gallic acid/g of dry weight, flavonoids equal to 5.45 ± 0.11 mg of quercetin/g of dry weight and ABTS value equal to 43.93 ± 2.58 mg Trolox equivalents/g of dry weight. These data confirmed that these by-products represent interesting raw materials from the chemical point of view [25,27], being characterized by a nutritional composition more interesting than fruit pulp [23] (Table 4). Coated and uncoated samples were very comparable the first day of storage. Similar low concentrations of TPC and TFC were recorded in both control and coated fruit, considering that, during extractions, the coating was not taken into account and the sole fruit pulp was used for chemical analyses. Samples were also comparable in terms of ABTS. The values of antioxidant activity were consistent with contents of TPC and TFC previously described, being phenol and flavonoid content precursors of antioxidant capacity [44]. As expected, values of polyphenols and flavonoids, as well ABTS data, dipped over time because various factors could affect their level in the peeled fruit, such as exposure of wounded tissue to light, air, enzymatic activity and chemical degradation [45]. Piga et al. [42] also verified that the polyphenols in minimally processed cactus pear declined significantly after six days of storage because polyphenols protected ascorbic acid and antioxidant activity from oxidation. Ochoa-Velasco and Guerrero-Beltrán [14], in their study dealing with fresh-cut cactus pears treated with acetic acid and chitosan, found a different trend for white and red prickly pears; in particular, while the phenolic content in white prickly pears slightly reduced, for red prickly pears, the phenolic content significantly (*p* < 0.05) increased. The two above authors justified the increase in the red cultivar with water loss [14]. Looking at data after 10 days of storage between coated and uncoated fruit, it was interesting to see a significant difference (*p* < 0.05) in terms of all tested chemical parameters. Specifically, the values in the control prickly pears were always higher than values recorded for the coated fruit. This experimental evidence was also recorded by Ochoa-Velasco and Guerrero-Beltrán [14], who applied acetic acid and chitosan to peeled fruit. A direct comparison among our data with other findings from literature cannot be easy because different analytical methods were used for determining the TPC content and the antioxidant activity. For example, Liguori et al. [22] found two different trends of antioxidant activity for uncoated and coated fruits stored at 5 °C for 9 days. In particular, these authors verified that the radical scavenging activity (DPPH) in control samples decreased during storage, showing a loss of 51% from the beginning to the end of the cold storage period. Otherwise, they verified that DPPH assay values in coated samples were almost stable during storage, showing values 2.1 times higher than in the controls at end of the cold storage period.

**Figure 4:**
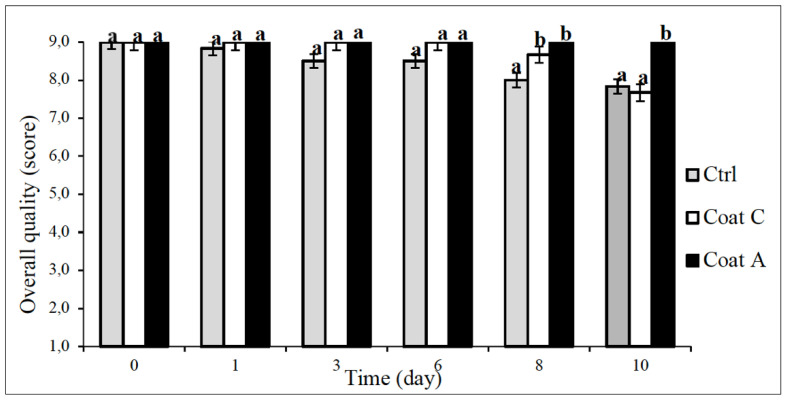
Sensory quality of control and coated prickly pears stored at 4 °C. Mean values ± standard deviation; Different letters indicate sample significantly different (*p* ˂ 0.05). Ctrl = sample without coating; Coat C = sample with alginate coating; Coat A = sample with alginate and peels.

Prickly pears were greatly appreciated for their sensory attributes by panel members during the entire observation period (Figure 4). As a fact, recorded scores ranged from 9 to about 7.5. Data in the graph refer to the overall quality of fruit but the trend of the data perfectly reflects the perception of each single sensory attribute considered to judge the product, i.e., color, odor and texture. All sensory parameters were highly prized during the first week of storage. Some small drops in the color scores were found for control and simply coated fruit in the last days of observation, whereas the prickly pears with peel-enriched coating always recorded the highest scores in all the sensory attributes.

This finding confirmed the ability of the fruit peel to better preserve product sensory quality during storage. The preservation action of prickly pear by-products on sensory quality was also assessed by Di Lucia et al. [33] who added fruit peels to fish burgers and verified that odor, color and texture in fortified samples were better retained, compared to control fish products, as both raw and cooked samples. The abundant literature available on the numerous active properties of these peels can justify the fact that peel addition to the coating was responsible for the better food quality recorded in the peel-enriched coated sample for the entire observation period [24,25,26,27].

The fitting of experimental data was also applied to sensory data to calculate the SAL values, i.e., the time within which the product remained acceptable in terms of sensory quality. The SAL values were found to be higher than 10 days, because all the samples never reached the threshold (score = 5). Considering that shelf life of peeled prickly pears depends on both microbiological and sensory quality evolution [15], data recorded from the fitting, in terms of both MAL and SAL, allowed defining the final product shelf life. Sensory quality did not represent a limiting factor for product acceptance because all the samples were greatly appreciated over time and no defects were highlighted by the panelists. Some problems appeared on minimally processed prickly pears in terms of microbial proliferation. As reported above, an undesired growth of mesophilic bacteria occurred after 4 days in the control fruit and after 6 days in both coated products (Table 1). Therefore, the shelf life of our ready-to-eat prickly pears was 4 days for uncoated fruit and about 6 days for coated products. Some better effects could be observed for the peel-enriched coated fruit, in terms of mold control and sensory quality preservation, thus suggesting that the use of peel powder in the coating could represent not only a sustainable way to recycle agri-food waste, but also a valuable approach to advantageously preserve product quality.

## 4. Conclusions

In this study the quality of coated and uncoated prickly pears was monitored during 10 days of refrigerated storage. Peeled fruits were coated with simple alginate or with alginate also containing prickly pear peel powder, to assess the effects of by-products on fruit quality throughout the storage period. Spoilage, pH and sensory properties of the fruits were properly investigated. Comparisons among the results highlighted differences between coated and uncoated fruit, thus confirming, on one hand, the already known effectiveness of the alginate-based coating, and, on the other hand, demonstrating that peels can further promote product preservation. In fact, if compared to the uncoated fruit, coated prickly pears presented lower weight loss and delayed microbial and fungal proliferation. When the two types of coating were compared, interesting findings were found in favor of the peel-enriched coating. Specifically, sensory quality was better retained when peels were used, due to the good preserving properties of this powder that contributed to a perfect maintenance of color, odor and texture for the entire observation period. Considering the great amount of waste that minimally processed prickly pears could generate, and the growing demand for ready-to-eat fruit, the approach adopted in this study, properly optimized, could gain important attention from the industrial sector that is very prone to finding more sustainable approaches for shelf life prolongation of fresh-cut produce.

## Figures and Tables

**Table 1:** Microbial acceptability limit (MAL) of coated and uncoated prickly pears during storage at 4 °C.

Samples	MAL (Day)			Microbiological Shelf Life (Day)
	MAL^TMB^	MAL^TPB^	MAL^Yeast^	
Ctrl Coat C Coat A	3.96 ± 0.37 ^a^ 6.41 ± 0.89 ^b^ 6.79 ± 0.52 ^b^	6.45 ± 0.66 >10 >10	7.3 ± 0.47 >10 >10	3.96 ± 0.37 ^a^ 6.41 ± 0.89 ^b^ 6.79 ± 0.52 ^b^

Mean values ± standard deviation; Means in the same column followed by different superscript letter are significantly different (*p* ˂ 0.05) MAL^TMB^ = MAL of Total Mesophilic Bacteria; MAL^TPB^ = MAL of Total psychrotrophic Bacteria; MAL^Yeast^ = MAL of Yeasts. Ctrl = sample without coating; Coat C = sample with alginate coating; Coat A = sample with alginate and peels.

**Table 2:** Molds in coated and uncoated prickly pears during storage at 4 °C.

Samples	Time (Day)					
	0	1	3	6	8	10
	Lactic Acid Bacteria					
Ctrl	1.60 ± 0.43 ^a^	4.08 ± 0.09 ^a^	5.50 ± 0.28 ^a^	5.13 ± 0.32 ^a^	5.30 ± 0.00 ^a^	7.70 ± 0.01 ^b^
Coat C	1.39 ± 0.55 ^a^	2.80 ± 0.28 ^b^	4.70 ± 0.50 ^b^	4.31 ± 0.19 ^b^	4.48 ± 0.52 ^b^	8.20 ± 0.28 ^a^
Coat A	1.00 ± 0.00 ^a^	3.56 ± 0.40 ^a^	3.17 ± 0.24 ^c^	4.31 ± 0.19 ^b^	4.69 ± 0.18 ^a,b^	8.00 ± 0.00 ^a,b^
	Molds					
Ctrl	2.0 ± 0.10 ^a^	4.48 ± 0.10 ^a^	4 ± 0.10 ^a^	4.24 ± 0.34 ^a^	5.50 ± 0.71 ^a^	6.45 ± 0.64 ^a^
Coat C	2.0 ± 0.10 ^a^	3.50 ± 0.71 ^a,b^	3.24 ± 0.34 ^b^	3.0 ± 0.10 ^b^	3.74 ± 0.37 ^b^	3.50 ± 0.71 ^b^
Coat A	2.0 ± 0.10 ^a^	2.5 ± 0.71 ^b^	3.0 ± 0.0 ^b^	3.15 ± 0.21^b^	3.50 ± 0.71 ^b^	3.65 ± 0.49 ^b^

Mean values ± standard deviation; Means in the same column followed by different superscript letter are significantly different (*p* ˂ 0.05). Ctrl = sample without coating; Coat C = sample with alginate coating; Coat A = sample with alginate and peels.

**Table 3:** The pH in coated and uncoated prickly pears during storage at 4 °C.

Samples	Time (Days)					
	0	1	3	6	8	10
Ctrl Coat C Coat A	5.76 ± 0.01 ^a^ 5.73 ± 0.00 ^a^ 5.70 ± 0.00 ^a^	5.58 ± 0.02 ^b^ 5.65 ± 0.01 ^a^ 5.72 ± 0.06 ^a^	5.42 ± 0.08 ^b^ 5.57 ± 0.01 ^a^ 5.61 ± 0.03 ^a^	4.92 ± 0.09 ^c^ 5.51 ± 0.02 ^a^ 5.34 ± 0.10 ^b^	4.89 ± 0.19 ^a^ 5.02 ± 0.21 ^a^ 4.82 ± 0.02 ^a^	5.37 ± 0.01 ^a^ 4.95 ± 0.01 ^b^ 5.19 ± 0.01 ^a^

Mean values ± standard deviation; Means in the same column followed by different superscript letter are significantly different (*p* ˂ 0.05). Ctrl = sample without coating; Coat C = sample with alginate coating; Coat A = sample with alginate and peels.

**Table 4:** Polyphenol content (TPC), flavonoid content (TFC) and antioxidant activity (ABTS) of coated and uncoated prickly pears during storage at 4 °C.

Sample	TPC		TFC		ABTS	
	Mg of Gallic Acid/g of Dry Weight		Mg of Quercetin/g of Dry Weight		Mg Trolox Equivalents/g of Dry Weight	
	t 0	t 10	t 0	t 10	t 0	t 10
Ctrl	1.86 ± 0.85 ^a^	1.31 ± 0.11 ^a^	0.92 ± 0.04 ^a,b^	0.69 ± 0.11 ^a^	4.55 ± 0.73 ^a^	3.53 ± 0.50 ^a^
Coat C	1.85 ± 0.30 ^a^	0.76 ± 0.11 ^b^	1.01 ± 0.02 ^b^	0.52 ± 0.06 ^b^	4.50 ± 0.64 ^a^	2.50 ± 0.31 ^b^
Coat A	1.57 ± 0.02 ^a^	0.86 ± 0.33 ^b^	0.89 ± 0.07 ^a^	0.47 ± 0.02 ^b^	3.80 ± 0.28 ^a^	2.24 ± 0.27 ^b^

Mean values ± standard deviation; Means in the same column followed by different superscript letter are significantly different (*p* ˂ 0.05). t0 = the initial sampling time; t10 = the sampling time after 10 days of storage; Ctrl = sample without coating; Coat C = sample with alginate coating; Coat A = sample with alginate and peels.

## Data Availability

Data presented in this study are available on request.
